# Computing infrared spectra of proteins using the exciton model

**DOI:** 10.1002/jcc.24674

**Published:** 2016-11-21

**Authors:** Fouad S. Husseini, David Robinson, Neil T. Hunt, Anthony W. Parker, Jonathan D. Hirst

**Affiliations:** ^1^ School of ChemistryUniversity of NottinghamNottinghamNG7 2RDUnited Kingdom; ^2^ Department of PhysicsUniversity of StrathclydeSUPA, 107 Rottenrow EastGlasgowG4 0NGScotlandUnited Kingdom; ^3^ STFC Rutherford Appleton LaboratoryCentral Laser FacilityHarwell CampusDidcotOX11 0QXUnited Kingdom

**Keywords:** two‐dimensional infrared spectroscopy, protein, molecular dynamics simulation

## Abstract

The ability to compute from first principles the infrared spectrum of a protein in solution phase representing a biological system would provide a useful connection to atomistic models of protein structure and dynamics. Indeed, such calculations are a vital complement to 2DIR experimental measurements, allowing the observed signals to be interpreted in terms of detailed structural and dynamical information. In this article, we have studied nine structurally and spectroscopically well‐characterized proteins, representing a range of structural types. We have simulated the equilibrium conformational dynamics in an explicit point charge water model. Using the resulting trajectories based on MD simulations, we have computed the one and two dimensional infrared spectra in the Amide I region, using an exciton approach, in which a local mode basis of carbonyl stretches is considered. The role of solvent in shifting the Amide I band (by 30 to 50 cm^−1^) is clearly evident. Similarly, the conformational dynamics contribute to the broadening of peaks in the spectrum. The inhomogeneous broadening in both the 1D and 2D spectra reflects the significant conformational diversity observed in the simulations. Through the computed 2D cross‐peak spectra, we show how different pulse schemes can provide additional information on the coupled vibrations. © 2016 The Authors. Journal of Computational Chemistry Published by Wiley Periodicals, Inc.

## Introduction

Understanding the three‐dimensional structure of a protein is often a challenging task but is an undertaking that can yield deep insights into biological functions, ranging from membrane signaling to catalysis to charge transfer as well as dynamic scaffolding, mechanical and electrical transduction. Approaches such as X‐ray crystallography and nuclear magnetic resonance can provide atomistic detail, while optical spectroscopy in the ultra‐violet and infrared (IR) regions can provide useful qualitative information. Efforts to understand and interpret the characteristic spectroscopic features of proteins have been ongoing for many decades. In the IR, the Amide I region lies between 1600 and 1700 cm^−1^,^1^, ^2^, ^3^ and is sensitive to the backbone conformation of a protein. This region has been extensively used to probe protein structure and dynamics, as it can provide useful information with respect to protein folding, misfolding, and unfolding.^3^, ^4^, ^5^, ^6^ However, the spectra often show convoluted and overlapping bands that can be challenging to decipher. There are distinctive spectral characteristics arising from α**‐**helices, β‐sheets, and random coil structures.^7^, ^8^, ^9^, ^10^, ^11^, ^12^ α**‐**helices exhibit a band between 1650 and 1658 cm^−1^.^3^ Bands near 1663 cm^−1^ have been associated with 3_10_ helices,^3^, ^7^, ^8^ while β‐sheets exhibit bands between 1620 and 1640 cm^−1^ as well as 1690 and 1695 cm^−1^.^3^, ^4^, ^5^, ^6^, ^7^

The characteristic vibrations of polypeptides in general consist of nine types: Amide A, B, and I–VII modes. The Amide I and II are interesting from a structural perspective as they give rise to two broad bands associated with the protein backbone. The former is the primary focus of this article. The Amide I mode is characterized by the C = O stretch which accounts for about 80% of the vibration, and the wagging and bending of the N—H bond which accounts for the remaining 20%.

Torii and Tasumi^13^ used *ab initio* calculations to investigate the *A* (singly degenerate) and *E* (doubly degenerate) components of the Amide I bands in the Raman and IR spectra of peptides. For helical conformations, the *A* component of the Raman band is intense and corresponds to the carbonyl groups vibrating in‐phase; the *E* component of the IR band is less intense whereby an out‐of‐phase vibrational combination leads to a net transition dipole moment perpendicular to the helix axis. For β‐sheets, the splitting of the characteristic intense peak at ∼1620 cm^−1^ and the weaker peak at ∼1690 cm^−1^ is directly proportional to the number of strands (up to a certain amount) in the sheet.^14^ For larger β‐sheets, the absorption becomes independent of the size of the sheet.

In the past 20 years or so, sophisticated experimental techniques have been developed to allow collection of IR spectra in two dimensions using both time (photon echo^15^) and frequency domain (double resonance^16^), methodologies.^16^, ^17^, ^18^, ^19^, ^20^, ^21^, ^22^, ^23^ Irrespective of the experimental approach, a 2D‐IR signal arises from a sequence of three laser‐sample interactions and the resulting spectrum is a correlation map of excitation and detection frequencies. This leads to the spreading of the molecular response over a second frequency axis, allowing resolution of features that are obscured by overlapping peaks in a traditional IR spectrum.

In a 2D‐IR spectrum, diagonal peaks represent signals featuring excitation (pump) and detection (probe) at the same frequency and these are analogous to the features observed in a 1D‐IR spectrum. Additional information arises from the 2D lineshape of these features, which reflects the temporal evolution of the local environment of a given oscillator. Off‐diagonal peaks arise when the excitation and detection frequencies differ and these provide insights into vibrational couplings, energy transfer or chemical exchange processes. The shape of these off‐diagonal peaks can also be influenced by coupling between vibrational modes.

2D‐IR has been increasingly applied to protein samples and a wide range of applications have been reported. These have included the spectroscopy and dynamics of disordered polypeptides,^24^, ^25^, ^26^ picosecond protein conformational dynamics,^27^, ^28^, ^29^, ^30^ amyloid fibril formation^31^, ^32^, ^33^, ^34^ and the structure of transmembrane proteins.^35^, ^36^, ^37^ These have been extensively reviewed elsewhere.^38^, ^39^, ^40^, ^41^

Fully *ab initio* or density functional theory (DFT) calculations of the vibrational frequencies of large polypeptides are currently prohibitively demanding of computational resources. Thus, more approximate approaches are adopted. Krimm and Bandekar^2^, ^3^ recognized that the nature of the Amide I band is influenced by the interactions of carbonyl vibrations via electrostatics and they constructed the Transition Dipole Coupling (TDC) model, which captures an essential part of the inter‐peptide couplings. The model has formed the basis for interpreting the Amide I bands of polypeptides in linear absorption spectra^42^ and has been extended to the analysis of 2D‐IR spectra. Hamm and Woutersen^43^ suggested a Transition Charge Coupling model that included higher order multipole contributions, and further improved on the TDC results. Although the model was, in general, consistent with DFT studies, it could not describe through bond coupling.

The influence of the molecular environment on individual local modes has attracted significant attention.^44^, ^45^, ^46^, ^47^, ^48^, ^49^, ^50^, ^51^, ^52^, ^53^, ^54^ Ham and Cho^47^ provided a framework for considering the influence of the electrostatic environment, with the development of coupling and frequency maps derived from *ab initio* calculations on model peptides such as *N*‐methylacetamide (NMA) and dipeptides. Both the coupling and frequency shift maps are dependent on the main chain dihedral angle of the dipeptide. These frequency maps can include the effects of water surrounding the chromophores as well as other components such as ions or lipids. Many of these maps have been developed over the past decade, some derived from *ab initio* calculations^50^, ^55^, ^56^, ^57^, ^58^, ^59^ and some, such as Skinner's,^60^ derived empirically. These maps have been widely adopted to calculate short‐range interactions, and are used in conjunction with the TDC model for the long‐range interactions.

Early^20^, ^61^ calculations of Amide I bands used models based on simple geometric properties relating to the nature of hydrogen bonding. Karjalainen et al.^54^ studied a set of 44 proteins, calculating Amide I spectra by empirically optimizing parameters in several terms accounting for the effects of solvent, the local environment, and inter‐peptide hydrogen bonding. Their work showed how the shift in frequency is strongly dependent on the number of hydrogen bonds to the amide oxygen atom or the amide NH group. This empirical approach contrasts with the more sophisticated models based on different electrostatic properties such as the electric field, electric field gradient, and the electrostatic potential.

Ganim and Tokmakoff^62^ examined the influence of conformational fluctuations on computed Amide I bands in 1D and 2D IR spectra for three small proteins using molecular dynamics (MD) simulations. The fluctuations of both the solvent and solute influenced the calculated IR lineshapes. They reported that the computed lineshapes were broader than the experiment. Choi et al.^63^ presented computational (semi‐empirical and MD) simulations and theoretically predicted the IR, 2D IR, electronic and vibrational dichroism spectra of ubiquitin. In their simulations, the backbone atoms were constrained to keep the conformations close to those obtained from semi‐empirical geometry optimizations. They found that hydration had a significant effect on the computed IR spectra, contributing to the computed red shift of the Amide I bond of different structural components. Recently, Jansen and coworkers have benchmarked several approaches to computing the Amide I band and 2D IR of proteins from MD simulations.^64^, ^65^ Up to four proteins were studied using several combinations of force fields for the MD simulations, electrostatic mappings and couplings. Skinner's empirical frequency map^60^ with the TDC model was reported to do well in conjunction with the OPLS‐AA force field. However, it was concluded that there is still considerable scope for understanding and improving modeling approaches. Our study provides some additional complementary insight into the current state of the art.

We develop further insight into the relationship between protein conformation and spectra. Using the electrostatic potential to compute the coupling and frequency maps, we consider a range of well‐studied, typical proteins and investigate how the environment can affect protein IR spectra in the Amide I region. We model the shift of spectral peaks resulting from the fluctuating surrounding environment with the aid of MD simulations. To investigate the effect of solvent and conformational dynamics on the Amide I spectra, we use a test‐bed of eight globular proteins^42^ plus ubiquitin.^63^ The former were previously used^42^ to study the effect that irregularities and distortions in structural components had on the Amide I bands. Following Grechko and Zanni,^66^ we investigate the influence of various structural and dynamical aspects on the location and intensities of bands of 2D signals.

## Methodology

### Exciton Hamiltonian

Exciton theory^67^ provides a framework for considering a large polymeric system. The vibrational exciton one‐quantum Hamiltonian is constructed based on a system of coupled local modes:

(1) $$\hat{H}=H_{0}+F-E_{0}$$

in which $\hat{H}$ is the Hamiltonian, *H*_0_ is the Hamiltonian of *N* non‐interacting peptide groups, *F* is the inter‐peptide potential, and *E*_0_ is the ground state energy. Hence, the Hamiltonian matrix consists of three types of element: the diagonal elements which correspond to the harmonic (central) frequency, the off‐diagonal nearest neighbor coupling (NNC) constants, and the other off‐diagonal elements which describe the through‐space interaction between local mode vibrations. The TDC approximation^2^, ^3^ calculates the latter elements as:

(2) $$f_{ij}=\frac{0.1}{\varepsilon }\times \frac{{\vec{\mu }}_{i}.{\vec{\mu }}_{j}-3\left({\vec{\mu }}_{i}.{\vec{\eta }}_{ij}\right).\left({\vec{\mu }}_{j}.{\vec{\eta }}_{j}\right)}{r_{ij}^{3}}$$

where *f_ij_* is the TDC, *ε* is the dielectric constant, ${\vec{\mu }}_{i}$ is the transition dipole moment for the Amide I mode located on peptide *i*. ${\vec{\mu }}_{j}$ is the transition dipole moment for peptide *j*, *r_ij_* is the separation of the dipoles between peptides *i* and *j*, *η_ij_* is the vector defining the separation between the *i* ^th^ and *j* ^th^ peptide.

**Figure 1:**
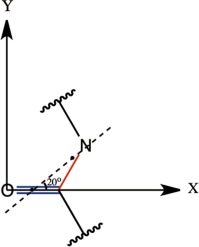
The location and orientation of the transition dipole. [Color figure can be viewed at wileyonlinelibrary.com]

Following Torii and Tasumi,^42^ the TDC was computed using a transition dipole (Fig. 1) placed 0.868 Å away from the amide carbonyl bond, and oriented 20**°** toward the amide nitrogen along the OCN plane. Its magnitude was 3.7 D Å^−1^ amu^−1/2^.

The nearest neighbor off‐diagonal Hamiltonian matrix elements are assigned from a NNC look‐up table or coupling map^57^ that consists of force constants calculated *ab initio* for all combinations of main‐chain dihedral angles (in increments of 30°) for a di‐peptide.

We now turn to the calculation (using a modified version of the dichrocalc software^68^) of the diagonal elements of the Hamiltonian and the change in frequency for each local mode due to the surrounding electrostatic environment. The electrostatic potential at site *i* is computed from a set of atom‐centered partial charges^47^:

(3) $$\phi_{i}=\sum_{j=1}^{N}\frac{c_{j}}{4\pi \varepsilon_{0}r_{i}{,}_{j}}$$

where *j* is the index that runs over all *N* partial atomic charges, *c_j_*, in the system. The atoms in the peptide group where the potential is calculated are excluded from the summation. The atomic partial charges for backbone atoms and side chains groups were taken from CHARMM36 force field.^69^ Explicit solvent (e.g., water) and hetero‐atoms are thus readily (and have been) included in the calculations. The interplay between the force field used to sample conformational dynamics and models used to construct the exciton Hamiltonian is complex.^64^, ^65^ Previous studies have considered CHARMM22^70^, ^71^ amongst other force fields. In our work, we use the CHARMM36 force field, where there is evidence^69^, ^72^ that changes in the internal parameters describing the peptide backbone give a better representation of the structure and dynamics than CHARMM22 as assessed through validation against various experimental (in many cases NMR) observables.

The electrostatic potential is used in conjunction with a combination of so called linear expansion coefficients to give the shifted frequency:

(4) $$\omega_{k}=\omega_{0}+\sum_{j=1}^{4}l_{j}\phi_{k,j}$$

where *ω_k_* is the shifted frequency of peptide *k*, *ω*_0_ is the central frequency (discussed later). Index *j* runs across the four atoms at which there are partial charges (Table 1) in each peptide *k*. The linear expansion coefficients *l_j_* (Table 1) are derived from the following equation:

(5) $$l_{j}=\;\frac{g^{I}}{4\pi cM_{I}^{2}{\left(\omega_{I}^{0}\right)}^{3}}{\left(\frac{\partial c_{j}}{\partial Q_{j}}\right)}_{0}^{eff}$$

where *g^I^* is the cubic anharmonic coefficient for the Amide I mode, *c* is the speed of light, *M_I_* is the reduced mass, $\omega_{I}^{0}$ is the angular frequency, and ${\left(\frac{\partial c_{j}}{\partial Q_{j}}\right)}_{0}^{eff}$ is the effective transition charge in units of e Å^−1^.

**Table 1:** The backbone partial charges from the CHARMM force field,^69^ assigned to each of the atoms in a peptide unit.

Atom	Partial charge (e)	*l_*j*_* (e)
C	0.51	0.00160
O	−0.51	−0.00554
N	−0.47	0.00479
H	0.31	−0.00086

Their respective linear expansion coefficients from the Cho^47^ map were used to calculate the shift in central frequency.

**Table 2:** Proteins studied with their PDB codes.

Protein	PDB Code	Class	% β‐strands	% β‐turns	% α‐helix	3_10_ ‐ helices	% Coil	No. solvent molecules
α‐Lactalbumin	1ALC	α + β	7	30	30	14	19	8053
Carbonmonoxy‐Myoglobin	1MBC	α	0	7	74	4	15	6860
Concanavalin A	3CNA	β	42	44	0	0	14	11227
Egg White Lysozyme	2LYM	α + β	6	40	30	10	14	8412
β‐Trypsinogen	2PTN	α + β	32	44	8	3	13	7641
Carboxypeptidase A	5CPA	α + β	16	30	35	2	17	10862
Ribonuclease A	7RSA	α + β	27	24	18	3	28	6379
Ubiquitin	1UBQ	α + β	32	28	16	8	16	5978
Flavodoxin	5NLL	α + β	21	19	37	4	19	6782

Their secondary structure content computed using PROMOTIF.^92^ The number of water molecules used in the MD simulations is provided.

The central frequency is usually chosen between 1650 and 1710 cm^−1^.^16^, ^73^, ^74^ We adopted a value of 1680 cm^−1^, which gives computed spectra consistent with the range observed experimentally for the Amide I region. The difference between this value and the gas phase value for NMA of 1717 cm^−1^ suggests that the electrostatic effect (as modeled here) does not fully account for the solvent‐induced shift. For proline residues, which do not have an amide hydrogen atom the frequency shift was not explicitly calculated; instead we simply used a fixed frequency of 1653 cm^−1^.^75^ Side chains that are known to absorb in the Amide I region such as those present in glutamine and asparagine have not been considered as chromophore groups; they are treated as side chains contributing to the electrostatic potential instead. The 1D absorption line spectra were convoluted with a Gaussian full width at half maximum bandwidth of 4 cm^−1^. This convolution accounts for broadening due to mechanisms not captured explicitly by the MD simulation. We illustrate how isotopic labeling of the carbonyl groups in different secondary structure elements in ubiquitin could be used to deconvolute the distinct contributions of helix and sheet to the 2D signal. The shift due to ^13^C^18^O isotope labeling lies between 60 and 75 cm^−1^.^50^, ^76^, ^77^, ^78^ We adopted a value of 65 cm^−1^ for residues belonging to secondary structure types of interest. To calculate the 2D spectra, the two‐quantum Hamiltonian is constructed from the one‐quantum Hamiltonian matrix elements as follows^62^:

(6) $$H_{m,n\;m,n}^{II}=H_{m,m}+H_{n,n}-\delta_{m,n}\Delta$$

(7) Hmm,nkmm≠nk, m≠kII=2Hm,kδm,n+Hm,nδm,k

(8) Hmn, nkm≠kII=Hm,kδm,n+δn,k

where *δ_m,n_* is Kronecker's delta, *H^II^* is the two‐quantum Hamiltonian, *H* is the one‐quantum Hamiltonian with site indices *m*, *n*, and *k*. Δ is the difference between the fundamental and overtone absorption frequencies, also known as the anharmonicity. The Hamiltonian operator for singly and doubly excited states can be expressed as^79^:

(9) H^=∑n=1Nεn|n〉〈n|+∑m,n=1NJmnm〉〈n+∑m,n=1Nεm+εn−Δδmn|mn〉〈mn|+∑m,n=1N∑j,k=1m,n≠j,kNJmn,jk|mn〉〈jk|

where *J* is the coupling constant between the singly excited |m〉, |n〉 states or doubly excited |mn〉 and 〈jk| states. *ε* is the site energy of the relevant state. *N* is the number of sites. The local transition dipoles $\vec{\mu }$, are likewise constructed from the one‐quantum transition dipole moments to produce two‐quantum transition dipole moments using the following expression:

(10) $${\vec{\mu }}_{m,n}=\sqrt{2}{\vec{\mu }}_{m}\delta_{m,n}+{\vec{\mu }}_{n}\left(1-\delta_{m,n}\right)$$

with *m* and *n* again being the site indices. The two‐exciton Hamiltonian matrix is diagonalized to produce a set of $\frac{N^{2}+N}{2}$ energies that are used to compute the non‐linear response. For a protein of *N* oscillators, there are *N* ^2^ two‐quantum states and the number of interactions (or matrix elements in the Hamiltonian) grows as *N* ^4^, which makes the calculations significantly more demanding than for 1D‐IR.

The third order non‐linear polarization *P* ^(3)^ is a convolution^80^, ^81^, ^82^, ^83^, ^84^ of the third order non‐linear response functions *R* ^(3)^ and the *three* electric fields *E_n_*:

$$P_{\left(t\right)}^{3}=\int_{0}^{\infty }dt_{3}\int_{0}^{\infty }dt_{2}\int_{0}^{\infty }dt_{1}E_{3}\left(t-t_{3}\right)E_{2}\left(t-t_{3}-t_{2}\right)$$

(11) $$E_{1}\left(t-t_{3}-t_{2}-t_{1}\right)R^{\left(3\right)}\left(t_{3},t_{2},t_{1}\right)$$

where *t_n_* refers to the time intervals between laser pulses. *R* ^(3)^ describes the macroscopic behavior of the system under the effect of the optical fields between the time intervals.

In 2D photon echo experiments, the diagonal peaks appear as positive signals while they appear as negative bleaches in double resonance experiments. The off‐diagonal contributions to the 2D signal; however are both positive and negative. We computed the two‐quantum Hamiltonian using a modified version of the Zanni and Hamm code^80^ reading into the *peptide.c* code the one‐quantum exciton Hamiltonians constructed for each snapshot, and uses a fixed anharmonicity of 16 cm^−1^. The Hamiltonian is diagonalized and the corresponding unitary transformation is used to transform the transition dipole matrix. The dipole approximation is used, whereby cross‐excitations are not allowed. The 2D signal is evaluated as the sum of the rephasing and nonrephasing components. The computed spectra shown in this article are purely in the frequency domain, and the diagonal and off‐diagonal contributions to the 2D signal shown here are positive and negative signals respectively (as in photon echo experiments). Thus the positive signal represents the ground state depletion (bleach) and stimulated emission ( v=0→1), while the negative signal corresponds to excited state emission ( v=1→2). Polarization conditions have been examined previously by Hochstrasser.^82^ The peak intensities are collected by ensemble averaging the lab frame dipole moment components to account for the orientation of residues with respect to the laser polarization. Our 2D signal is computed using the *ZZZZ* polarization condition:

(12) $$〈Z_{\alpha }Z_{\beta }Z_{\gamma }\left(Z_{\delta }\right\rangle 〉=\frac{1}{15}\left(〈\cos\theta_{\alpha \beta }\cos{\;\theta }_{\gamma \delta }〉+〈\cos\theta_{\alpha \gamma }\left(\cos\theta_{\beta \delta }\right\rangle 〉+\;〈\left\langle \cos\theta_{\alpha \delta }\cos\theta_{\beta \gamma }\right)〉\right)$$

where *θ*_mn_ is the angle between transition dipoles *m* and *n*. In a later section, we show the enhancement of cross‐peaks by subtracting two computed spectra: one using the *ZZZZ* condition and the other using the *ZXXZ* pulse condition.^63^, ^80^, ^81^, ^82^, ^83^ Similar to eq. (12), the latter pulse condition can be expressed as:

(13) $$〈Z_{\alpha }X_{\beta }X_{\gamma }\left(Z_{\delta }\right\rangle 〉=-\frac{1}{30}\left(〈\cos\theta_{\alpha \beta }\cos\left({\;\theta }_{\gamma \delta }\right\rangle 〉-4〈\cos\theta_{\alpha \gamma }\left(\cos\theta_{\beta \delta }\right\rangle 〉+\;〈\left\langle \cos\theta_{\alpha \delta }\cos\theta_{\beta \gamma }\right)〉\right)$$

The enhanced signal is computed using:

(14) $$Sig_{en}=〈\left(ZZZZ\right\rangle 〉-3〈\left(ZXXZ\right\rangle 〉$$

### Molecular dynamics simulations

Using the NAMD 2.9 molecular dynamics package,^84^ we performed MD simulations on ubiquitin and the eight proteins studied by Torii and Tasumi.^42^ The structures were taken from the Protein Data Bank, and the N‐ and C‐termini were capped to give ( ${\text{NH}}_{3}^{+}$‐C_α_H_2_) and (‐CH_2_‐ ${\text{CO}}_{2}^{-}$), respectively. For the cases of α‐lactalbumin, concanavalin A and myoglobin, the *apo* forms of the proteins were used. Neutralization was achieved by adding 9 Na^+^ ions for α‐lactalbumin, 9 K^+^ for concanavalin A, 16 K^+^ for flavodoxin, 2 Cl^‐^ for myoglobin, 5 Cl^‐^ for ribonuclease A, 6 Cl^‐^ for trypsin, and 8 Cl^‐^ for lysozyme. The simulations included explicit water to model the influence of conformational dynamics on line broadening, and to investigate the effect of solvation on the Amide I band. Each protein was solvated in a hexagonal prism of TIP3P water molecules,^85^ and periodic boundary conditions were applied. To account for long‐range interactions, the Particle Mesh Ewald method^86^ was used, and the Lennard‐Jones cutoff was 12 Å. Energy minimization was performed for each protein for 30,000 cycles.

Thereafter an equilibration process with an integration time‐step of 2 fs ran for 0.5 ns, during which all covalent bonds involving hydrogen were constrained using the SHAKE algorithm.^87^ Experiments are usually performed in deuterated water. The water molecules in our simulations have rigid O—H bonds. Thus, the TIP3P model, captures hydrogen bonding and electrostatic effects but, neither the influence of the vibrations of water nor the effect of the deuteration on the conformational dynamics of the protein are considered.

Production dynamics were performed for a period of 2 ns in the NPT ensemble using Langevin dynamics and a damping coefficient of 5 ps^−1^. The Nosé–Hoover^88^, ^89^, ^90^ and Langevin piston^91^ periods were set to 100 fs and their time‐decay period was set to 50 fs to keep the temperature constant at 300 K, while maintaining pressure at 1 atm. Snapshots were sampled uniformly every picosecond. Trajectory files with and without solvent were saved separately to investigate the effect of solvent on the computed spectra. The 2 ns trajectories are short, but the main purpose is to provide a sample of configurations close to the experimental structures. Our unconstrained simulations will potentially explore a broader and more physical range of equilibrium conformations than the constrained simulations of Choi et al.^63^

## Results and Discussion

**Figure 2:**
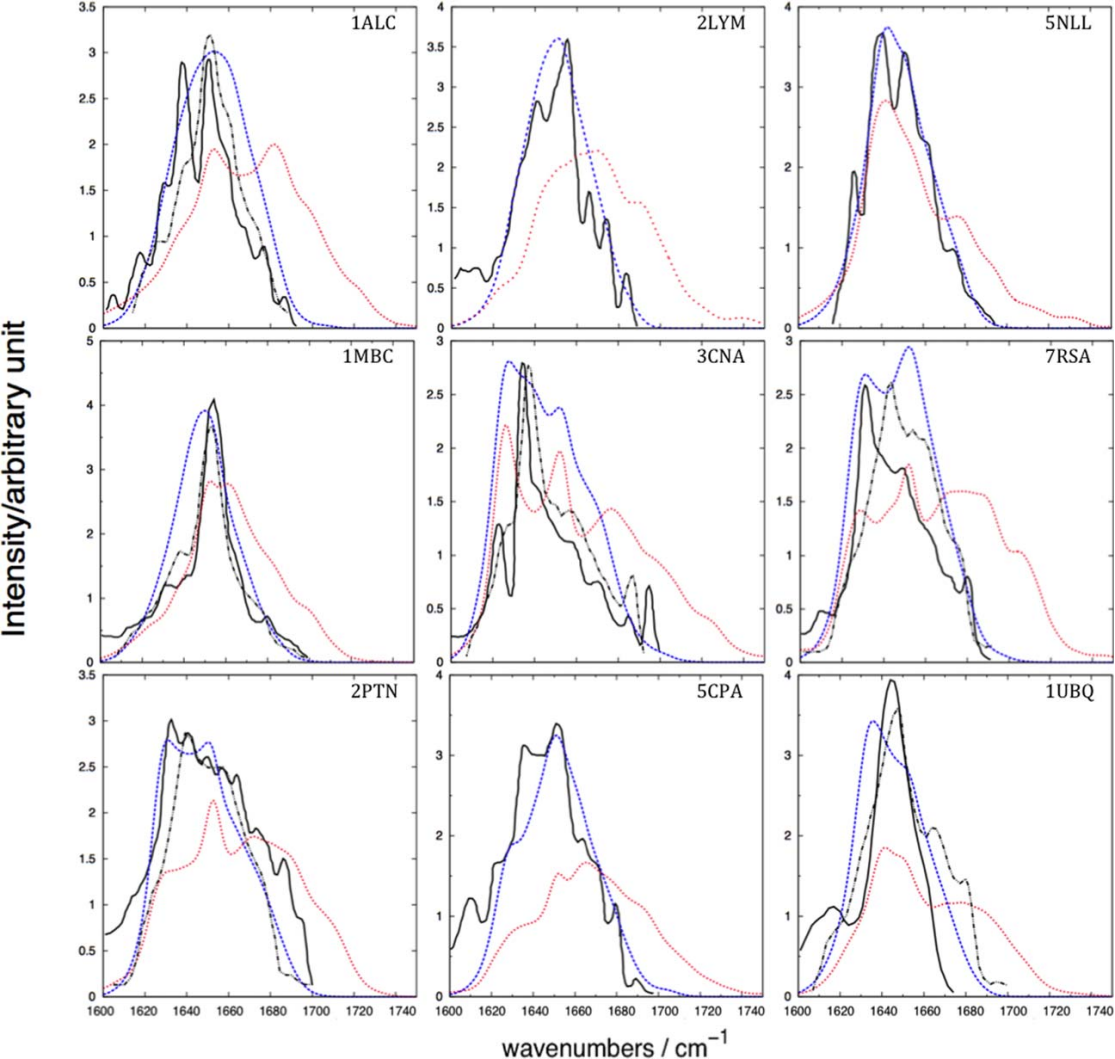
Amide I IR spectra of the nine proteins. The solid black line represents the experimental spectra from various sources cited by Torii and Tasumi, J. Chem. Phys., 1992, 96, 3379, reproduced by permission,^42^ who reported that the spectra were “weakly deconvoluted”. The dashed black represents the experimental (recorded for 3% protein solution in H_2_O) transmission IR spectra taken from Karjalainen et al., J. Phys. Chem. B, 2012, 116, 4831, reproduced by permission.^54^ The dashed blue represents the average computed spectra including solvent, and the dotted red line represents the average computed spectra excluding solvent. See Table 2 for the names of the proteins. [Color figure can be viewed at wileyonlinelibrary.com]

Experimental transmission IR spectra were taken from the literature^42^, ^63^ rescaled such that the highest intensity peaks match the computed spectra, and plotted against the computed spectra of the nine proteins (Fig. 2). The experimental conditions used for recording the spectra cited by Torii and Tasumi^42^ were as follows: α‐lactalbumin and lysozyme were recorded using a 3.5% protein solution in D_2_O; myoglobin and trypsin using a 5% protein solution in H_2_O, ribonuclease A using a 10% protein solution in H_2_O, while spectra for carboxypeptidase A, concanavalin A, and flavadoxin spectra were all recorded using a 5% protein solution in D_2_O. We present our computed spectra without any post‐processing to enhance the fine structure. The overall band shape of each of the computed spectra for solvated proteins agrees with the experiment (Fig. 2).

**Figure 3:**
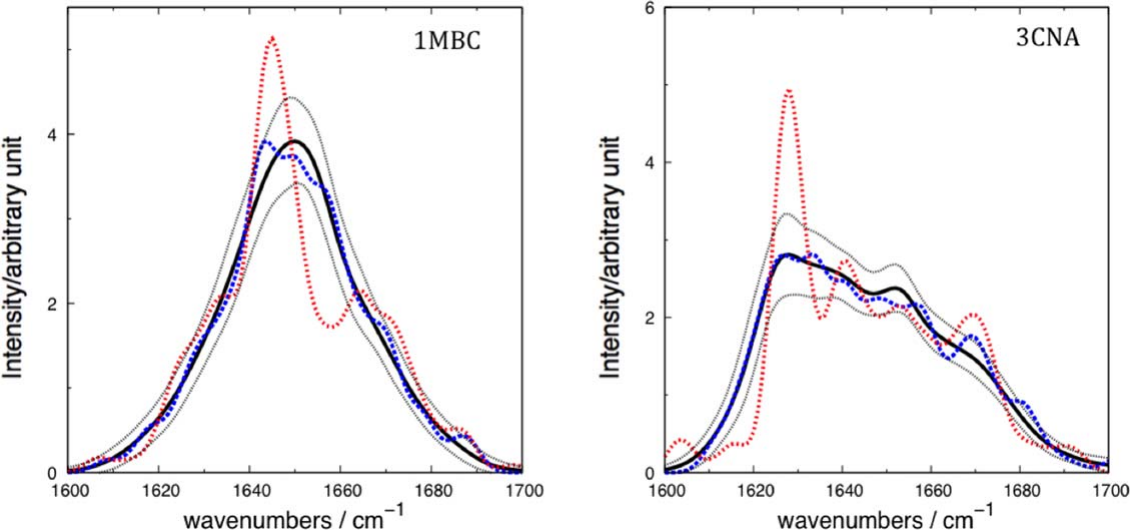
Computed spectra for a) α‐helical protein carbonmonoxy‐myoglobin (1MBC) and b) β‐sheet protein concanavalin A (3CNA), for the snapshots with spectra most similar (dashed blue), and least similar (dotted red) to the computed average spectra (solid black). The thin dotted lines (upper and lower) represent the mean average ± standard deviation, respectively. [Color figure can be viewed at wileyonlinelibrary.com]

The spectra computed neglecting the solvent exhibit an Amide I band that is broader than the experiment, extending beyond 1700 cm^−1^ to around 1750 cm^−1^. We believe that neglecting solvent in the calculations means that the surface residues have an environment that is artefactually more different from the buried residues compared to the situation for a solvated system. To investigate the influence of conformational diversity on the computed spectra, we calculated the standard deviation of the computed intensity at each wavenumber over all snapshots (Fig. 3). We also identified the individual snapshots giving rise to the computed spectra that were least and most similar to the mean computed spectra over the 2 ns simulation period (Fig. 3).

**Figure 4:**
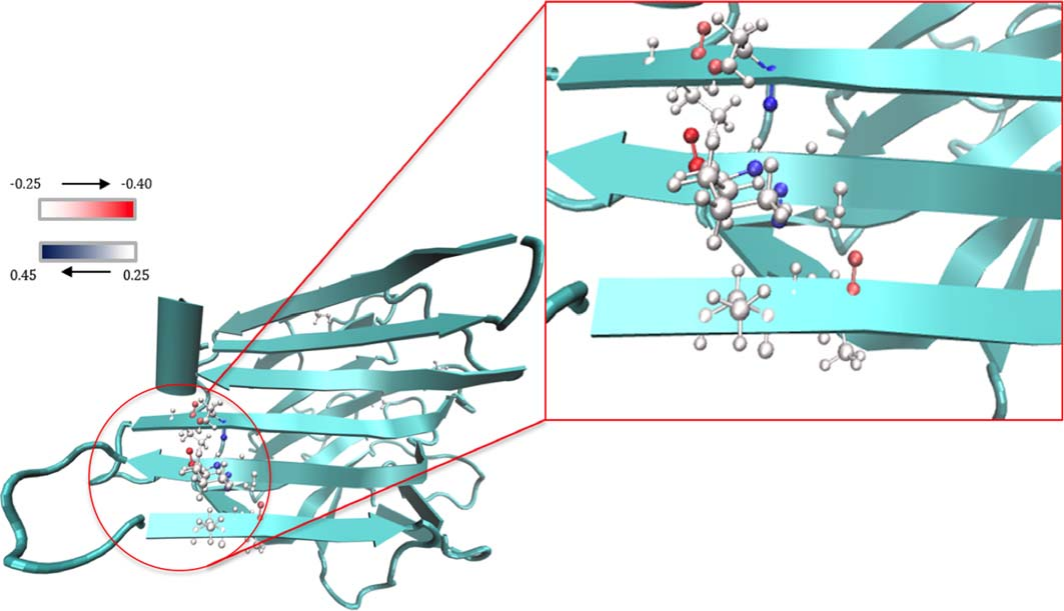
Concanavalin A in one of the snapshots of interest. The strongly coupled residues are depicted with an atomic representation. The color bar scale represents the eigenvector coefficients: red indicates a negative coefficient, blue a positive one and the atoms are colored accordingly. [Color figure can be viewed at wileyonlinelibrary.com]

The spectra from individual snapshots that are most dissimilar to the ensemble show some of the most intense features. By examining these conformations, we can characterize the extent of (de)localization of the vibrations associated with the most intense features. The squares of the eigenvector coefficients reflect the contributions of the local modes to the transition.^93^, ^94^ For the most dissimilar snapshot of concanavalin A, only two coefficients had a squared magnitude greater than 0.25, that is, none of the transitions was particularly localized. Of particular note was a pattern observed near the end of the 2 ns trajectory. The intense peak was located between 1622 and 1626 cm^−1^. In this spectral region of the simulation, the vibration was delocalized across different residues in parallel strands. This is consistent with the strength of the through‐space coupling constants between these residues. Figure 4 shows the location of the vibration in the context of the protein structure. The coupling between the residues fluctuates over the simulation, but certain conformations (Fig. 4) exhibited strong inter‐strand coupling between residues perpendicular to the strand orientation of the sheet, which is consistent with the findings of Woys et al.^95^

**Figure 5:**
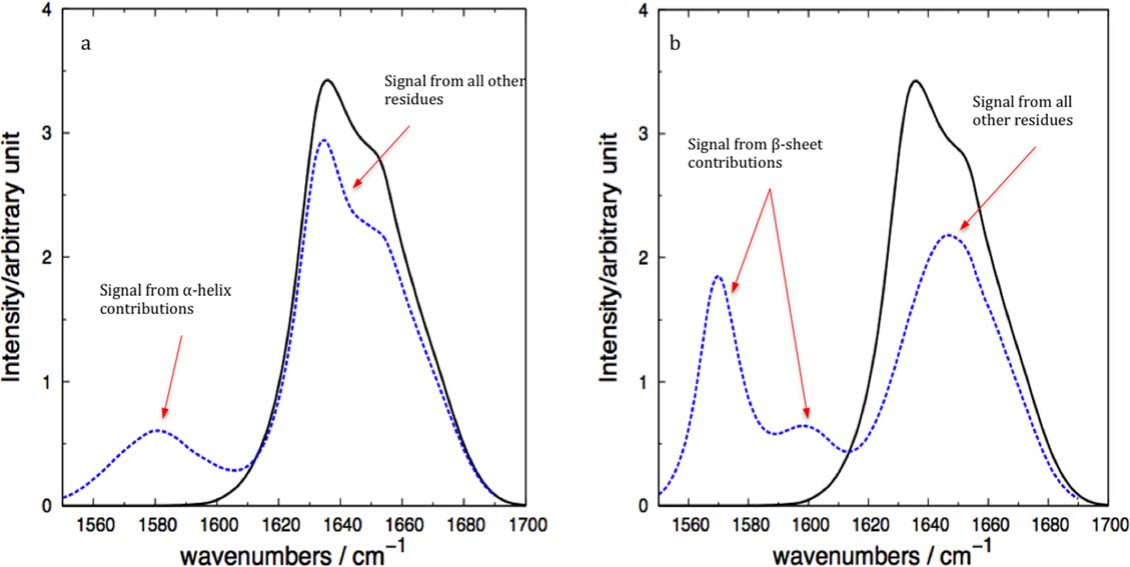
Computed 1D spectra of ubiquitin (1UBQ) with ^13^C^18^O isotope labeling of the α‐helix residues a), and β‐sheet residues b). The solid black line in both panels represents the unlabeled computed spectrum, while the dashed blue line represents the isotope labeling cases. [Color figure can be viewed at wileyonlinelibrary.com]

Experimentally,^96^, ^97^ it is possible using expressed protein ligation and native chemical ligation to isotope label distinct regions in proteins, for example, specific elements of secondary structure. The computational analogue is readily performed and can help us to understand the various contributions to the spectra and deconvolute the overlapping bands of the 1D signal. We illustrate this for the simulations of solvated ubiquitin. The result of isotope labeling of either residues in α‐helices or in β‐sheets (Fig. 5) shows a shift in peaks accordingly. The residues belonging to α‐helices show a contribution to the 1D signal in the form of a single broad peak at 1580 cm^−1^, while the contribution from β‐sheet residues shows two peaks, one more intense than the other. Splitting the signal by isotope labeling has an effect on the overall band maxima, which will be discussed later.

**Figure 6:**
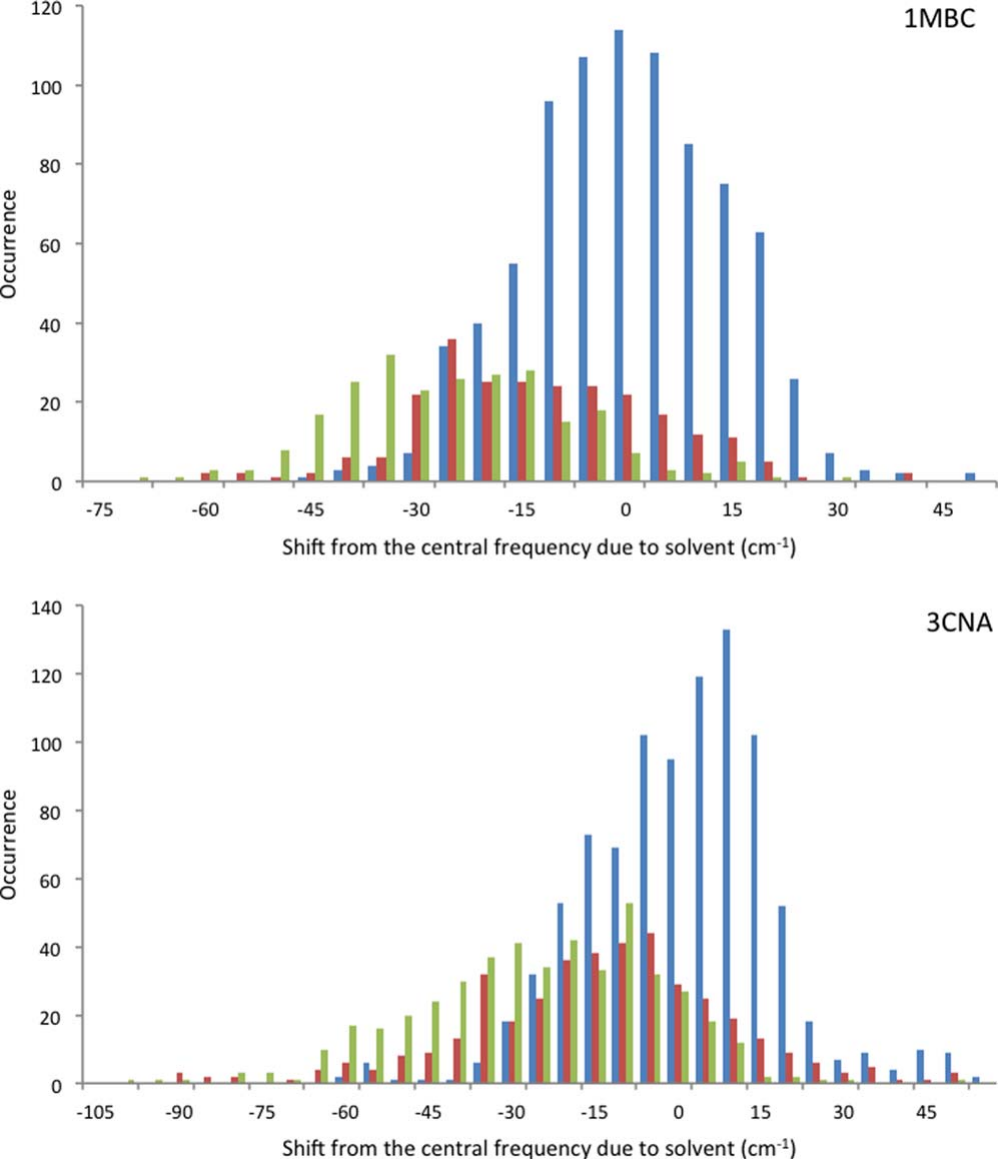
Histograms for carbonmonoxy‐myoglobin (1MBC) and concanavalin A (3CNA), depicting the relationship between the number of hydrogen bonds to solvent and the shift in intrinsic frequency due to solvent [i.e., eq. (3) evaluated considering just the water molecules as the environment]. The blue line represents the case of no hydrogen bonding occurring between the carbonyl oxygen and solvent molecules, the red represents the case where there is one hydrogen bond, and green represents the case where there are two. The histograms were computed using 10 snapshots, evenly distributed across the first nanosecond of the MD simulation. [Color figure can be viewed at wileyonlinelibrary.com]

Karjalainen et al.^54^ assumed the shift in intrinsic frequency is related to the presence or absence of a hydrogen bond between a carbonyl oxygen of one amide group and an amide hydrogen of another. We investigated the relationship between shift in intrinsic frequency and the presence of hydrogen bonds to solvent. The relationship is evident, but appears to be a weak one (Fig. 6). For example, in the case of the α‐helical carbonmonoxy‐myoglobin, the number of hydrogen bonds to solvent molecules shows a modest influence on the extent of the negative shift from the central frequency, as is expected. Concanavalin A, a β‐sheet protein, shows a weaker but similar trend. Our modeling of the influence of hydrogen bonds is through the electrostatic potential, which is a non‐local approach. It may be that a more explicit treatment of hydrogen bonding would identify a stronger relationship between hydrogen bonding and the frequency shift.

### 2D IR spectra

**Figure 7:**
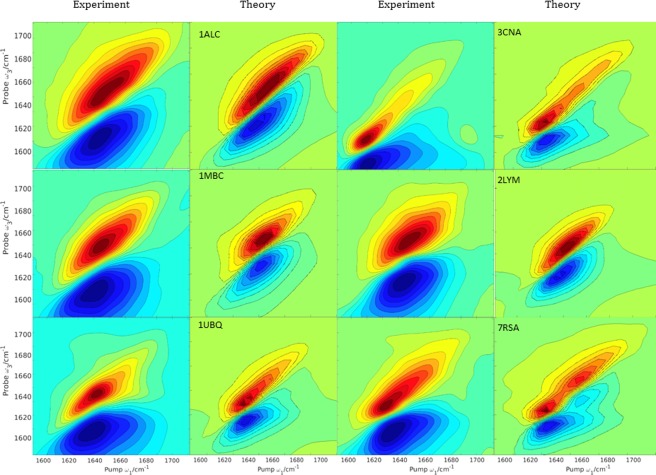
Experimental^102^ and computed 2D absorptive spectra using the *ZZZZ* scheme for: α‐lactalbumin (1ALC), carbonmonoxy‐myoglobin (1MBC), ubiquitin (1UBQ), concanavalin A (3CNA), lysozyme (2LYM), and ribonuclease A (7RSA). Time delay (*t*_2_) in both the experimental and computed spectra was zero. The contours for the computed spectra are plotted with a 10% intensity of the maximum amplitude with 20 uniformly spread contours from the minimum to the maximum intensity. [Color figure can be viewed at wileyonlinelibrary.com]

Conformational dynamics of the protein and solvent are reflected in the 2D lineshapes. We have computed the absorptive 2D IR spectra for ubiquitin, concanavalin A, carbonmonoxy‐myoglobin, lysozyme, ribonuclease A, and α‐lactalbumin based on the conformations sampled from the MD trajectories. We chose these proteins as a benchmark due to their different sizes and mixed structural compositions. For ubiquitin, we investigated the convergence of the computed spectra with the number of conformations sampled from the MD trajectory. Spectra computed with 200 snapshots sampled uniformly across the trajectory gave a computed spectrum very similar to that computed with 2000. So the 2D‐spectra for the larger proteins were computed with 200 snapshots to limit the computational cost. Thus inhomogeneous broadening in the calculated 2D spectra is a result of the structural changes depicted by the snapshots, and homogeneous broadening was modeled by convoluting with a 2D Lorentzian bandwidth of 10 cm^−1^. The vibrational motional narrowing effect, whereby the line width may be overestimated by static structural snapshots,^98^, ^99^, ^100^ may influence the computed spectrum. Whilst this is a significant effect for NMA in solution,^49^ it may be less important for polypeptides, because of the spread of the multiple amide modes of the protein.^101^ Different features can be seen in the spectra (Fig. 7). The intense peaks which are plotted with pump frequency, *ω*_1_, on the horizontal axis and the probe frequency, *ω*_3_, on the vertical axis as in Ref. 102 correspond to contributions from the α‐helices [*ω*_1_, *ω*_3_] = [1635, 1635] cm^−1^ in the case of ubiquitin. For concanavalin A, the anti‐parallel sheet contribution can be seen as a weak peak appearing at [1660, 1660] cm^−1^. Two intense signals are also seen between [1625, 1625] cm^−1^, both associated with out‐of‐phase oscillations of carbonyls of the same β‐strand.^74^ A broad peak stretches along the diagonal from [1620, 1620] cm^−1^ to [1670, 1670] cm^−1^. This agrees with experiment^102^ and is associated with random coils in concanavalin A. Random coils probably contribute to the weaker signal at [1660, 1660] cm^−1^.

In all spectra shown in Figure 7, the elongation along the diagonal stretches from 1620 to 1670 cm^−1^ and shows that the solvent‐exposed residues experience a fluctuating electrostatic environment, due to conformational disorder and fluctuations of the solvent. The spectrum of carbonmonoxy‐myoglobin is dominated by contributions from α‐helices. A broad and intense signal stretches from [1640, 1640] to [1655, 1655] cm^−1^. This is also seen for α‐lactalbumin [1630, 1630] to [1660, 1660] cm^−1^. Table 3 summarizes the location of the peak centers in the computed and the experimental spectra of Figure 7. Overall, our computed spectra agree well with the experimental spectra. The level of agreement is comparable with recently reported calculations^64^, ^65^ using different force fields and modeling protocols.

**Table 3:** Locations of diagonal and off‐diagonal peaks in the computed and experimental^102^ 2D‐IR spectra.

	Diagonal peak locations (*ω*_1_ = *ω*_3_)/cm^−1^	
Protein	Experiment	Computed
1ALC	1645	1650
1MBC	1650	1647
1UBQ	1640	1635
2LYM	1640	1635
7RSA	1640	1640
3CNA	1620	1625
	Off‐diagonal peak locations (*ω*_1,_ *ω*_3_)/cm^−1^	
Protein	Experiment	Computed
1ALC	(1640,1610)	(1640,1630)
1MBC	(1640,1610)	(1640,1635)
1UBQ	(1640,1610)	(1630,1625)
2LYM	(1640,1620)	(1630,1620)
7RSA	(1640,1620)	(1620,1620)
3CNA	(1620,1600)	(1625,1620)

**Figure 8:**
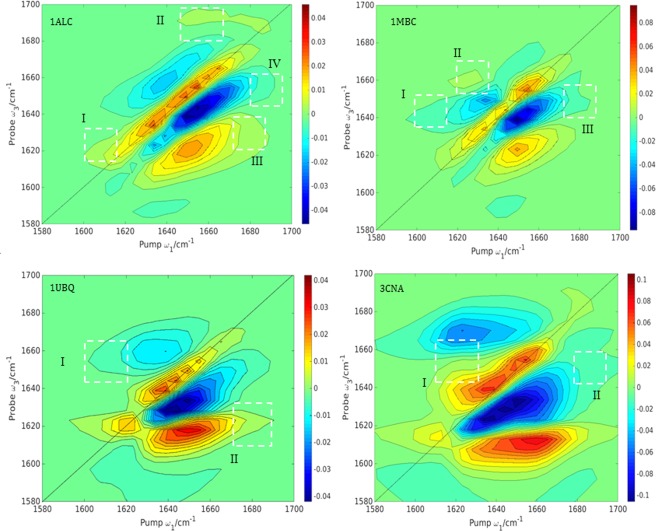
Computed <*ZZZZ*>−3 < *ZXXZ*> cross‐peak 2D IR absorptive spectra computed for α‐lactalbumin (1ALC), myoglobin (1MBC), ubiquitin (1UBQ), and concanavalin A (3CNA). Cross‐peaks are highlighted with white dotted squares. The contours are plotted with a 10% intensity of the maximum amplitude with 20 uniformly spread contours from the minimum to the maximum intensity. [Color figure can be viewed at wileyonlinelibrary.com]

More detailed information can be accessed by enhancing the cross‐peaks (Fig. 8). For example, coupled vibrations of different transitions are now revealed compared to the *ZZZZ* spectra. The cross‐polarization $〈\left(ZZZZ\right\rangle 〉-3\left\langle 〈ZXXZ〉\right)$ signal suppresses the diagonals to some extent and enhances other features of the 2D spectra. The off‐diagonal peaks are better resolved than their counterparts in Figure 7, allowing the nature of the coupling between different local modes to be explored further. Much weaker peaks appear on the diagonal in Figure 8 compared to the intense diagonal peaks in Figure 7. These weak diagonal peaks (Fig. 8) are now comparable in intensity with the off‐diagonal cross‐peaks, and these features merge somewhat to give a broad peak which extends parallel to the *ω*_1_ axis, as can be seen for example in the case of concanavalin A: peak I, Figure 8. The splitting between the positive and the negative signals of a cross‐peak is a measurement of the coupling strength and is due to the off‐diagonal anharmonicity. For example in concanavalin A, peak I indicates there is a strong coupling between structural elements, while in the rest of the proteins the analogous peak indicates weaker coupling. Table 4 summarizes the cross‐peaks highlighted in Figure 8.

**Table 4:** The cross‐peaks in the cross‐polarization 2D‐IR spectra (Fig. 8) for α‐lactalbumin (1ALC), carbonmonoxy‐myoglobin (1MBC), ubiquitin (1UBQ) and concanavalin A (3CNA).

Protein	Peak (strength)				Structural components involved			
1ALC	I (w)	II (w)	III (w)	IV (w)	β	α + β	β	β + coil
1MBC	I (w)	II (w)	III (w)		α	α	α + coil	
1UBQ	I (w)	II (w)			α	β		
3CNA	I (s)	II (w)			β	β		

The coupling is characterized as either strong (s) or weak (w).

In the cross‐polarization spectra of α‐lactalbumin and myoglobin (Fig. 8), there are signs of contribution from coupled unstructured coils. Unstructured coils appear as broad featureless peaks at [*ω*_1,_ *ω*_3_] = 1650 or 1660 cm^−1^,^79^ and the cross‐polarization spectra reveal broad peaks stretching from 1660 cm^−1^ toward 1680 cm^−1^ due to β‐strands coupled with unstructured coils (in the case of α‐lactalbumin: peak IV), and coupled unstructured coils with β ‐turns (in the case of myoglobin: peak III). The shape of the two cross peaks is different and the finding agrees with a previous study^103^ to suppress random coil features.

Cross‐peaks usually come in pairs, but when one half of the doublet is more intense (as is the case in peak IV for lactalbumin and myoglobin), it generally means that the off‐diagonal anharmonicity is weak between the two local sites.^80^ The splitting between the diagonal and off‐diagonal peaks is underestimated in Figure 7, which may suggest that the value of the anharmonicity used in the calculations should be greater. Choi et al.^63^ and Chung et al.^104^ previously investigated ubiquitin in two separate studies. Their spectra exhibited a Z‐shape, due to the contribution from anti‐parallel β‐sheet strands. We find a similar *Z*‐shape in both our ubiquitin and concanavalin A crossed‐polarization spectra (Fig. 8).

**Figure 9:**
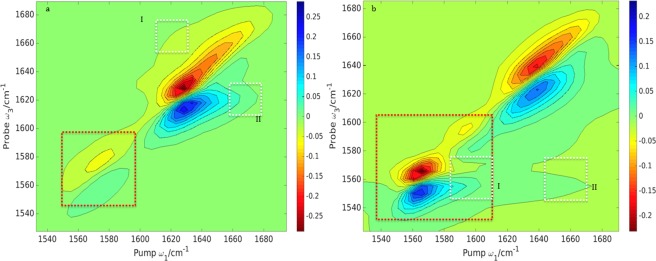
Computed 2D absorptive spectra using the *ZZZZ* scheme of cases where the α‐ helices a) and β‐strands b) of ubiquitin (1UBQ) are isotope labeled. The contours are plotted with a 10% intensity of the maximum amplitude and 20 uniformly spread contours from the minimum to the maximum intensity for the left panel and 20 similar uniformly spread contours for the right panel. The isotope labeled components are highlighted with red dotted squares and the cross peaks with white dotted ones. [Color figure can be viewed at wileyonlinelibrary.com]

The crossed‐polarization spectroscopy is clearly a powerful, experimentally realizable approach, which provides an additional insight into the origins of the 2D signal. Some of these origins can also be explored using different computational strategies. We investigate the contributions to the 2D signal by isotope labeling the α‐helices and β‐strand secondary structures of ubiquitin. Figure 9 shows the two cases when either the helices or the sheets are labeled.

**Figure 10:**
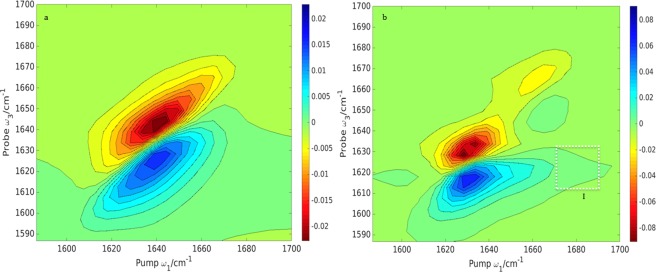
Computed 2D absorptive spectra using the *ZZZZ* scheme of α a) and β b) secondary structure separately of ubiquitin (1UBQ). The contours are plotted with a 10% intensity of the maximum amplitude and 20 uniformly spread contours from the minimum to the maximum intensity for the left panel and 20 similar uniformly spread contours for the right panel. Cross‐peaks are highlighted with white dotted squares. [Color figure can be viewed at wileyonlinelibrary.com]

Both isotope labeled components have been shifted to ∼1570 cm^−1^ and are consistent with the computed 1D spectra (Fig. 5). The diagonal peak intensities for the labeled α‐helices are clearly much weaker than those for the rest of the protein. This is more evident in the 2D spectra as the signal is proportional to the fourth power of the transition dipole moment, whereas in the 1D spectra, the intensity depends on the square of the transition dipole moment. The cross‐peak at ∼ [1660, 1560] cm^−1^ in Figure 9b indicates coupling between β‐sheet and random coil components. The absence of an analogous cross‐peak in Figure 9a indicates there is little or no coupling between β‐sheet and α‐helices. We have computed the 2D spectra of the α‐helices and β‐sheets separately (Fig. 10).

There are 12 residues in α‐helices and 26 residues in β‐strands. Two Hamiltonians were constructed in which the size of the one‐quantum Hamiltonian was 12 × 12 in the first case and 26 × 26 in the second, with all elements in the environment: main chain, side chains, and solvent molecules contributing to the electrostatic potential and shift in frequency. Figure 10 shows the contributions to the signal from both α‐helices and β‐strands. The β‐sheet spectrum is about four times more intense than the α‐helices spectrum consistent with the 2:1 ratio observed for the 1D spectrum. The contribution from the α‐helices is a diagonal elongated peak with its center at ∼[1640, 1640] cm^−1^ while the β‐strands contribute three peaks: two intense ones at ∼[1625,1625] and ∼[1638, 1638] cm^−1^ and a weak one at ∼[1660, 1660] cm^−1^ indicating coupled local modes. There is also a weak cross‐peak at [1680, 1620] cm^−1^ (Peak I). The presence of Peak I in Figures 10b and Peaks I and II Figure 9a is further indication of the coupling between β‐secondary structure components.

### Transition dipole strengths

Non‐linear spectroscopies are more sensitive to transition dipole strengths than linear spectroscopies. The 2D intensities scale as ${\left|\vec{\mu }\right|}^{4}$ compared to 1D intensities that scale as ${\left|\vec{\mu }\right|}^{2}$. Grechko and Zanni^66^ studied the 1D and 2D IR of a model α‐helical system and concluded that the transition dipole strength of the random coil state is 0.12 D^2^, and that a greater magnitude indicates a more ordered system with vibrational excitonic states forming associated with secondary structure. Here, we extend that consideration to six of the proteins, extracting the two‐quantum transition dipole moments that contribute to the 2D diagonal signal. In Table 5, we compare the per residue contribution to the 2D intensities, ${\left|\vec{\mu }\right|}_{\frac{total}{residue}}^{4}$ for the aforementioned proteins. The absolute value of the transition dipole, ${\left|\vec{\mu }\right|}_{total}^{4}$ was computed as a sum over all transition dipole moments. One might anticipate that more ordered structures such as concanavalin A should give more delocalized excitons, which should be manifested in more intense bands.

**Table 5:** The studied proteins (with PDB codes), the number of residues, and fraction of coil composition, the two‐quantum transition dipole strengths per residue ${\left|\vec{\mu }\right|}_{\frac{total}{residue}}^{4}$.

Protein	Number of residues	_% Coil residues_	${\left|\vec{\mu }\right|}_{\text{total}/\text{residue}}^{4}\left(D^{4}\right)$	${\left|\vec{\mu }\right|}_{\text{total}/\text{residue}}^{2}\left(D^{2}\right)$
α‐lactalbumin (1ALC)	122	19	0.47	0.69
Myoglobin (1MBC)	152	15	0.47	0.69
Lysozyme (2LYM)	128	14	0.40	0.63
Concanavalin A (3CNA)	237	14	0.80	0.89
Ubiquitin (1UBQ)	76	16	0.32	0.57
Ribonuclease A (7RSA)	123	28	0.48	0.69

The fraction of coil composition was computed from PROMOTIF,^92^ as in Table 2.

The per residue contribution, ${\left|\vec{\mu }\right|}_{\frac{total}{residue}}^{2}$ which is computed here by taking the square root of ${\left|\vec{\mu }\right|}_{\frac{total}{residue}}^{4}$, confirms delocalized excitonic vibrations as expected from ordered structured systems, and all the values fall above the threshold observed by Grechko and Zanni.^66^ However, there is not an obvious relationship between the proportion of coil structure in the protein and the intensity of the 2D diagonal signal. This discrepancy might be explained by the fact coil structures in folded proteins do not necessarily have the same properties as random coils in unfolded peptides such as the ones studied by Grechko and Zanni.^66^

## Conclusion

Overall, the calculations have quantitatively reproduced 1D‐IR spectra for the proteins studied. We have investigated the sensitivity of the Amide I peaks to conformational dynamics. Our motivation was to investigate the effect of conformational dynamics on the overall lineshapes. The mixing of the local modes that underlies the 1D‐IR spectra is revealed more explicitly, although not with complete clarity in the 2D‐IR spectra. Our study shows that the Cho model^47^ in combination with MD simulations confirm the importance of conformational dynamics in affecting the overall absorbance. We would expect different maps^50^, ^55^, ^56^, ^57^, ^58^, ^59^, ^60^ would yield qualitatively similar findings, although some of the quantitative detail might vary.^62^, ^105^

The computed 2DIR spectra for ubiquitin, carbonmonoxy‐myoglobin, α‐lactalbumin, concanavalin A, flavadoxin, and ribonuclease A are broadly in agreement with that presented in previous studies.^62^, ^63^, ^102^, ^104^ Solvation plays a significant role in the nature of the computed spectra. Through calculations of the Amide I band and analysis of the exciton Hamiltonian matrix, it is possible to relate specific conformational features to the IR spectrum. Delving deeper into the dynamics of proteins and studying single snapshots for both concanvalin A and carbonmonoxy‐myoglobin provides a stepping stone to understanding the appearance of the 2D spectra. The width of the 1D spectra is reflected in the linewidth of the diagonal on the 2D spectra. The off‐diagonal cross peaks were enhanced for ubiquitin, carbonmonoxy‐myoglobin, α‐lactalbumin, and concanavalin A by computing the $\left\langle 〈ZZZZ〉\right)-3\left\langle 〈ZXXZ〉\right)$ spectra for each case. We explored whether there is a relationship between the contribution to the 2D diagonal signal and the fraction of random coil, but none was evident. We are currently studying the dynamics and 2D IR spectroscopy of protein‐ligand binding, and investigating the behavior of coupled modes during binding and dissociation.
